# The role of electrical coupling in the decision to initiate swimming in young frog tadpoles

**DOI:** 10.1186/1471-2202-13-S1-P58

**Published:** 2012-07-16

**Authors:** Michael Hull, David Willshaw, Alan Roberts

**Affiliations:** 1 Institute for Adaptive and Neural Computation, University of Edinburgh, EH8 9AB, UK; 2 School of Biological Sciences, University of Bristol, BS8 1UG, UK

## 

Most neuronal networks are very complex and detailed information on the neurons and their connections is lacking. To investigate the significance of electrical coupling it is therefore necessary to find simpler networks for study. At hatching, *Xenopus laevis* tadpoles show two modes of locomotion; fictive swimming and struggling, making it an interesting model animal for studying relatively simple network dynamics[1]. Experiments have shown that fictive swimming can be generated by a central pattern generating (CPG) network of approximately 2000 neurons in the caudal hindbrain and the rostral spinal cord[1]. The neurons can be distinguished into groups, based on anatomy, morphology and firing characteristics[1]. During swimming, a particular group of neurons with descending axons (dINs) are the first neurons to fire on each side and act as 'trigger' neurons; synchronizing firing in each side of the CPG[2]. These neurons are electrically coupled, probably via gap junctions between their axons. Blocking this coupling makes the generation of swimming unreliable[3]. Since it is only possible to record from a limited number of neurons simultaneously and dINs are electrically coupled, electrophysiological investigation into dINs is difficult. Modelling allows us to test hypotheses and ask questions which are experimentally impossible.

Tadpoles respond to stimulation to the head by starting to swim. Recent work (Buhl et al. submitted) has defined a three step pathway from the headskin to the CPG network. The headskin is innervated by trigeminal afferent neurons. These synapse onto a population of ~20 trigeminal interneurons (tINs), which then synapse onto the population of dINs in the hindbrain. In life, in response to stimulation, the tadpole will either not react or will start swimming. We show that the electrical coupling plays an important role in converting a continuously variable input signal (headskin stimulation) into a discrete decision (initiation of swimming).

We first built multi-compartmental models of an individual dIN with an axon using Hodgkin-Huxley style current models from voltage-clamp data. Next, a model of a column of 30 dINs was built, with an anatomically realistic layout of somata and axons in the hindbrain. We investigated biologically feasible mechanisms for distributing gap junctions between the axons, which produced coupling coefficients between neurons similar to those measured physiologically. Next, a model of the synaptic input from the tINs onto the dIN network was built, converting a 'stimulation level' into a set of input spike times. We show that the presence or absence of electrical coupling plays an important role in the dynamics of group recruitment; with electrical coupling, increasing the chemical synaptic input to the network in small steps leads to an abrupt switch in behaviour, from few dINs firing to the entire population firing.

Modelling was done in a high-level Python API for simulating small networks of multi-compartmental neurons, using the NEURON simulator as a backend[4].

Using the one-sided model as a foundation; we will explore a fuller, bilateral model of the CPG; in particular investigating the roles of reciprocal inhibition, background NMDA excitation, and electrical coupling on the initiation of swimming in the tadpole.

## References

[B1] Roberts A Li W-C Soffe SR ‘How neurons generate behaviour in a hatchling amphibian tadpole: an outline’ Front. Behav. Neurosci 2010 4 16 20631854 10.3389/fnbeh.2010.00016 PMC2903309

[B2] Soffe SR Roberts A Li W-C ‘Defining the excitatory neurons that drive the locomotor rhythm in a simple vertebrate: insights into the origin of reticulospinal control’ J. Physiol 2009 587 20 4829 4844 10.1113/jphysiol.2009.175208 19703959 PMC2770150

[B3] Li W-C Roberts A Soffe SR ‘Locomotor rhythm maintenance: electrical coupling among promotor excitatory interneurons in the brainstem and spinal cord of young Xenopus tadpoles’ Journal of Physiology 2009 587 1677 1693 10.1113/jphysiol.2008.166942 19221124 PMC2683956

[B4] Hines ML Carnevale NT Expanding NEURON's Repertoire of Mechanisms with NMODL Neural Computation 2000 12 995 1007 10.1162/089976600300015475 10905805

